# Prevalence of oral mucosal lesions in human immunodeficiency virus‐infected children attending the Pediatric Infectious Diseases Clinic in Cape Town

**DOI:** 10.1002/cre2.484

**Published:** 2021-09-29

**Authors:** Riaan Mulder, Nadia Mohamed, Olorato Mathiba

**Affiliations:** ^1^ Restorative Dentistry The University of the Western Cape Cape Town South Africa; ^2^ Pediatric Dentistry The University of the Western Cape Cape Town South Africa

**Keywords:** highly active antiretroviral therapy, HIV, mucosal disease, oral manifestations

## Abstract

**Objective:**

Investigation of the prevalence of oral mucosal lesions in human immunodeficiency virus (HIV)‐infected children undergoing highly active antiretroviral therapy (HAART).

**Materials and Methods:**

Cross‐sectional study of 66 HIV seropositive children, comprised of 28 (42.4%) females and 38 (57.6%) males (average age of 6 years). Study participants all required data regarding CD4+ T‐helper cell counts and the viral load. All participants underwent an orofacial clinical examination by calibrated clinicians. Associations between the presence of oral mucosal lesions, CD4+ cell counts, and viral load were analyzed using Poisson regression.

**Results:**

The prevalence of oral manifestations was detected in 21 children (31.8%). Oral lesions were detected in 16 children with viral load copies <50 cells/mm^3^ and 22 children with CD4+ counts >500 cells/mm^3^. Predominant lesions identified included angular cheilitis (36.7%), candidiasis (13.3%) and atypical oral ulcers (13.3%). The presence of one lesion was the most prevalent represented by 19 children. Oral lesions in relation to the CD4+ counts >500 resulted in; 14 children with one oral lesion, 5 with two lesions and 3 with three oral mucosal lesions. The other half of this CD4+ count patient group presented with no oral mucosal lesions. Oral lesions in relation Viral load copies <50 resulted in; 9 children with one oral lesion, 3 with two oral mucosal lesions and 4 with three oral mucosal lesions. The other half of this Viral load patient group presented with no oral mucosal lesions. No significant correlations were established between the presence of oral mucosal lesions and low CD4+ counts (*p* = 0.715) nor with high viral load counts (*p* = 0.638).

**Conclusion:**

HIV‐related oral mucosal lesions still presented in the participants despite management with HAART. Based on the results, CD4+ counts and viral load does not appear to be suitable markers of orofacial involvement in children.

## 1 INTRODUCTION

Human immunodeficiency virus (HIV) infections are characterized by suppressed immune systems. The virus targets CD4+ T‐helper cells (CD4+ cells) in the immune system, which are meant to assist the body in fighting infections. The virus replicates within these CD4+ cells until it eventually destroys them (Calles, Evans, & Terlonge, 2010); this results in a decreased amount of CD4+ cells and an increased viral load (Wilson et al., 2008). Globally, 160,000 new HIV‐infected children cases are recorded every year; this resulted in 940,000 children between the ages of 0 and 14 years receiving antiretroviral therapy in 2018 (UNAIDS & Barton‐Knott, 2019). The 2012 HIV/AIDS prevalence report, concerning South African children between the ages of 0 and 14 years, stated that 1.25 million children were infected out of a population of 52.8 million people (Shisana et al., 2014). In 2017, the same age group represented 3 million infected children out of a total South African population of 57 million people (Simbayi et al., 2018). Furthermore, a South African study recorded an oral lesion prevalence of 51.8% among children between the ages of 0 and 14 years. Interestingly, the study's statistical analysis found no association between age and the prevalence of oral mucosal lesions (Duggal, Abudiak, Dunn, Tong, & Munyombwe, 2010). Moreover, several cardinal oral lesions are associated with the disease progression of HIV/AIDS, including oral candidiasis, hairy leukoplakia, Kaposi sarcoma, linear gingival erythema, necrotizing ulcerative gingivitis, necrotizing ulcerative periodontitis, and non‐Hodgkin's lymphoma (NHL) (Coogan, Greenspan, & Challacombe, 2005). Oral manifestations of HIV/AIDS are among the earliest signs of the disease and may function as markers of disease progression in both adults and children (Coogan et al., 2005; Miziara & Weber, 2008).

Normal CD4+ cell counts for adolescents and adults range between 500 and 1200 cells/mm^3^ (Bofill et al., 1992). Furthermore, while infants and young children normally possess higher CD4+ counts than adolescents or adults, they slowly decline to adult values by the age of 6 years (Shearer et al., 1997). Subsequently, this signifies a need to consider age when dealing with the immunological status of children, especially because the percentages of CD4+ cell counts are imperative when determining the immunological staging in children. The viral load is a blood test that determines the concentration of HIV copies in a person's blood plasma and is expressed as copies/ml or as a log_10_ value (Wilson et al., 2008). Clinicians use this test's results to assess the severity of an infection. Ideally, the viral load of a patient on antiretroviral medication should either remain undetectable or manifest below the detection limit of 50 copies/ml (Doyle et al., 2012). A rising viral load is often indicative of drug failure (Bofill et al., 1992). This is alarming because a decrease in CD4+ cells and an increased viral load make the body more susceptible to opportunistic infections (Wilson et al., 2008; Yengopal, Bhayat, & Coogan, 2011). Some of these infections result from commensal microorganisms that take advantage of the immunosuppression and manifest as different infections, including fungal, viral, and bacterial infections as well as neoplastic lesions (Coogan et al., 2005; Yengopal et al., 2011). These opportunistic infections often manifest in the oral cavity.

Nevertheless, the relevant literature asserts that a CD4+ count on its own is not a reliable marker for HIV progression in pediatric cases (Ramos‐Gomez et al., 1996), which has led to the inclusion of oral mucosal lesions. These oral mucosal lesions are determined in the cited Table 1 of the reference article on orofacial lesions associated with pediatric HIV infection (Ramos‐Gomez et al., 1999). The inclusion of oral mucosal lesions in the WHO clinical staging of HIV/AIDS has significantly benefited developing countries, especially those with poor resource settings where access to laboratory services is limited and turnaround times for blood tests are longer than usual (Yengopal, Kolisa, Thekiso, & Molete, 2016). Therefore, the presence of these oral mucosal lesions acts as clinical signposts concerning the presence and progression of a patients' immunodeficiency (Baghirath, Krishna, Gannepalli, & Ali, 2013; Yengopal et al., 2016). Additionally, oral manifestations have been reported as the earliest indicative signs of a pediatric HIV infection (Ramos‐Gomez et al., 1999); therefore, they aid in determining the prognosis and progression of an HIV infection (Coogan et al., 2005). However, although the utilization of oral lesions as predictors regarding the progression of HIV infections in adults has been thoroughly investigated and documented, there is a dearth of data concerning its efficacy in pediatric populations (dos Santos Pinheiro et al., 2009; Meless et al., 2014; Oladokun, Okoje, Osinusi, & Obimakinde, 2013; Rwenyonyi et al., 2011).

**Table 1:** The frequency distribution of children according to their age, gender, antiretroviral status, CD4+ cell count, viral load, and number of mucosal lesions

Characteristics	Number of children (% of children)	Number of mucosal lesions			
Gender		0	1	2	3
Male	38 (57.6%)	14	13	5	6
Female	28 (42.6%)	18	6	4	0
Age group (years)					
2–6	34 (51.5%)	18	7	5	4
7–12	32 (48.5%)	14	12	4	2
Antiretroviral status					
On HAART	63 (95.5%)	31	19	8	5
Not on HAART	3 (4.5%)	1	0	1	1
CD4+ count (cells/mm^3^)					
Unknown	14 (21.2%)	6	4	3	1
<200	3 (4.5%)	2	0	1	0
200–500	5 (7.5%)	3	0	0	2
500	44 (66.6%)	22	14	5	3
Viral load (copies/ml)					
<50	31 (47%)	15	9	3	4
50–1000	18 (27.3%)	10	6	2	0
1000–10,000	8 (12.1%)	4	1	3	0
>10,000	9 (13.6%)	4	2	1	2

The oral health status and prevalence of oral mucosal lesions among the children living with HIV/AIDS admitted to the Tygerberg Pediatric Infectious Diseases Clinic (Tygerberg PIDC) were unknown (Mohamed, Mathiba, & Mulder, 2020). This study aimed to investigate the array of oral mucosal lesions associated with HIV among children living with the disease.

## 2 MATERIALS AND METHODS

The study was conducted at the Tygerberg PIDC, which conducts antiretroviral therapy. It used a cross‐sectional descriptive study design in conjunction with the convenience sampling of candidates who fit certain inclusion criteria. Participant inclusion criteria included the following: children between the ages of 2 and 12 years with a confirmed HIV‐positive status; signed consent forms from legal guardians; recorded viral loads or CD4+ count result records at the time of the dental examination; and the ability to comply with a clinical examination conducted by the principal researcher (O. M.). An appropriate sample size was determined with a power calculation, and 50 patients were found to be adequately representative of the study population. The clinical team consisted of two dentists; the calibration of the principal researcher (O. M.) was conducted by the study leader (R. M.) and exhibited a Cohen's kappa of 0.92.

A quantitative data collection process was employed for the CD4+ counts and viral loads of the patients. The examinations inspected the patients' facial, extra‐oral, and intra‐oral tissues and included both an inspection and palpation of their mouths and facial structures. The soft tissue oral lesions included in this study consisted of the orofacial lesions that were outlined by the diagnostic criteria set out in the consensus classification of orofacial lesions associated with pediatric HIV infection (Ramos‐Gomez et al., 1999). To facilitate the presumptive diagnoses of lesions, two references served as pictorial lesion comparisons: a visual reference chart for health care workers (Wilson et al., 2008) and the reference consensus article (Ramos‐Gomez et al., 1999), which depicts common oral mucosal lesions in children with HIV/AIDS.

The data were entered into a Microsoft Excel 2010 spreadsheet. Subsequently, the statistical analysis was conducted using the R‐project program (Statistical analysis with R Core Team (2013); (R: A language and environment for statistical computing. R Foundation for Statistical Computing, Vienna, Austria).

**Figure 1:**
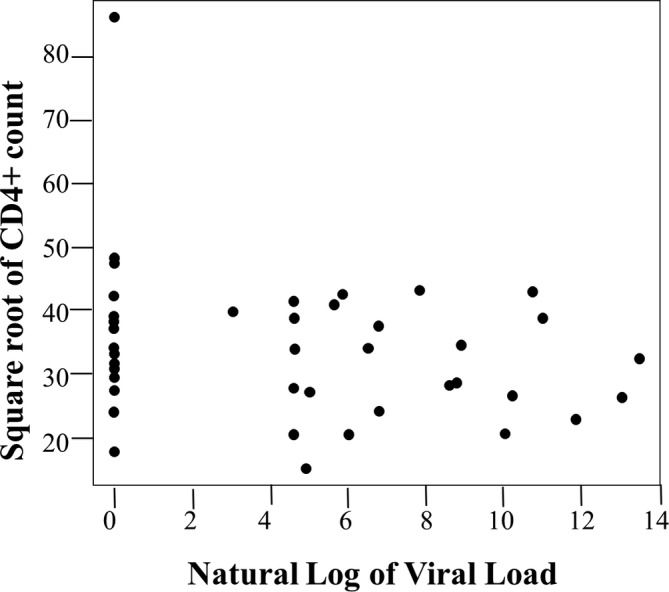
Association between CD4+ count and viral load

**Figure 2:**
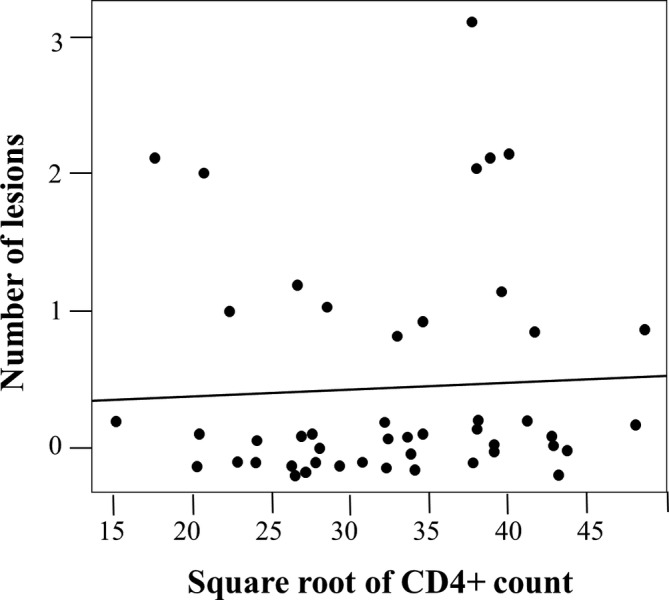
Association between the number of oral lesions and CD4+ count

**Figure 3:**
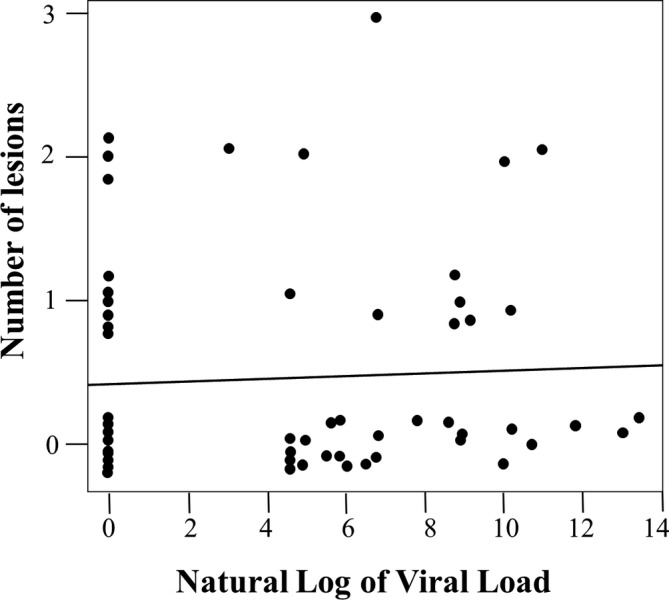
Association between the number of oral lesions and viral load

Although the data distribution of the CD4+ counts and viral loads was skewed, it was expected as it often occurs in HIV research. Therefore, a statistical calculation was conducted to achieve an informative plot for the collected CD4+/viral load data transformation. A natural log was used as the log of the statistically transformed variables of the CD4+ counts and the viral loads (Figure 1). The generalized linear models approach of the Poisson family was used to determine the association between oral mucosal lesions with the CD4+ counts (Figure 2) and oral mucosal lesions with viral load (Figure 3) that the children exhibited. Additionally, frequency distribution was used to summarize and identify the children's most common oral mucosal lesions and characteristics (Table 1).

The principal researcher (O. M.), who is a qualified dental practitioner, performed a standardized clinical examination on all the participants. A basic examination pack was used for the examination of each child; it consisted of an explorer probe, an intra‐oral mirror, gauze, and an LED headlight. All of the patients' oral mucosal lesions were recorded, and a data capture sheet documented the information obtained during the clinical assessment. The enrolled patients' data capture sheets were kept safely in a locked cabinet in the Principal Researcher's office.

Although children up to the age of 16 years are admitted to this clinic, only children between 2 and 12 years of age—with a confirmed HIV‐positive status—were enrolled in the study because the upper age limit for pediatric dental patients at the adjacent Tygerberg Oral Health Centre is 12 years (Mohamed, Mathiba, & Mulder, 2020). Regardless, following good clinical practice, brief oral health advice was given to all the children and caregivers. Furthermore, participating in the study was entirely voluntary, and participants were allowed to withdraw at any time. The written consent forms were thoroughly explained and issued to each participant's guardian. To maintain the confidentiality of the participants' identities in this study, the patients' names were not recorded in the data capture sheets. Instead, the patients' medical record numbers were used for identification purposes.

## 3 RESULTS

From the 300 patients treated at Tygerberg PIDC, this study recruited 66 children; (28 [42.4%] females and 38 [57.6%] males; the M:F ratio was 1.36:1) who complied with the inclusion criteria. Most participants (95.5%) were on highly active antiretroviral therapy (HAART). Nearly half of the patients (n = 31) on HAART presented with no oral mucosal lesions. The presence of one lesion was the most prevalent represented by 19 children. When the oral lesions in relation to the CD4+ counts >500 were considered; 14 children had one oral lesion, 5 children two lesions and 3 children presented with three oral mucosal lesions. The other half of this CD4+ count patient group presented with no oral mucosal lesions. When the oral lesions in relation to the Viral load copies <50 were considered; 9 children with one oral lesion, 3 with two oral mucosal lesions and 4 with three oral mucosal lesions. The other half of this Viral load patient group presented with no oral mucosal lesions. (Table 1). Ten of the patients presented with positive diagnoses of Tuberculosis and eight had oral mucosal lesions. The other co‐morbidities could not be inferred of their additional influence on the oral mucosal lesions as there was one patient per co‐morbidity. The recorded co‐morbidities included diabetes, Down syndrome, aplastic anemia, nephrotic syndrome, encephalitis, upper or lower respiratory tract infections and cytomegalovirus.

Figure 1 displays a plotted graph of the square root of the CD4+ count versus the natural log of the Viral Load. According to this graph, there is little to no correlation between the two variables; the Pearson correlation coefficient between them was −0.264, *p* = 0.073. Furthermore, the study compared the mean CD4+ counts and mean viral loads between children with and without oral mucosal lesions. This showed that, despite removing a clear outlier in the data, the correlation coefficient between them was −0.232, *p* = 0.121. Consequently, the difference between the children with oral mucosal lesions and the children without oral mucosal lesions was not statistically significant; that is, *p* = 0.701 and *p* = 0.176, respectively.

Figure 2 displays a plot concerning the number of lesions versus the square root of the CD4+ count. By fitting a linear regression with lesions designated as the dependent variable and CD4+ counts as the predictor, it was revealed that the effects of the CD4+ counts were not significant; *p* = 0.715. The generalized linear model approach, family = Poisson, was used for this analysis. It clearly illustrated a lack of correlation between the two variables. Notably, the lesion points were slightly offset from the integer values to make all the observations visible.

Figure 3 displays a plotted graph concerning the number of lesions versus the natural log of the viral load. The line on this graph represents the fitted model; it employs the same technique used for the lesions versus viral load comparison. The line's slope is not significantly different from zero, indicating a non‐significant association between the two variables; *p* = 0.638.

The results concerning the various oral mucosal lesions illustrated that Candidiasis infections were encountered most often, accounting for 44.4%. Regarding the prevalence of the oral manifestations associated with HIV, 21 children (31.8%) presented with one or more oral lesion (95% CI: 21.9–44.4), at the time of examination, predominantly comprising of Angular cheilitis (n = 11; 36.7%), Candidiasis (n = 4; 13.3%) and Atypical oral ulcers (n = 4; 13.3%). 35 (53%) of the children had mucosal lesions, with 19 (28.8%) presenting with one lesion.

Tables 2 and 3 display the relationships between various oral mucosal lesions and the patients' CD4+ counts and viral loads respectively. 66.6% of the lesions were present in children with CD4+ counts above 500 cells/mm^3^.

**Table 2:** Distribution of oral manifestations in relation to the CD4+ count in cells/mm^3^

Lesion type	>500 number of children (%)	500–200 number of children (%)	<200 number of children (%)	Unknown number of children (%)
Candidiasis:				
Pseudomembranous	2 (6.7)	0	0	1 (3.3)
Erythematous	0	1 (3.3)	0	0
Angular cheilitis	8 (26.7)	2 (6.7)	1 (3.3)	0
Parotid enlargement	0	0	0	1 (3.3)
Herpes labialis	3 (10)	0	0	0
Linear gingival erythema	1 (3.3)	0	0	1 (3.3)
ANUG	1 (3.3)	0	0	0
Atypical oral ulcers	1 (3.3)	0	0	3 (10)
Molluscum contagiosum	2 (6.7	0	0	1 (3.3)
Non‐Hodgkin's lymphoma	0	0	0	1 (3.3)
Total	18 (60)	3 (10)	1 (3.3)	8 (26.7)

**Table 3:** Distribution of oral manifestations in relation to the viral loads in copies/ml

Lesion type	<50 number of children (%)	<1000 number of children (%)	1000–10,000 number of children (%)	>10,000 number of children (%)
Candidiasis:				
Pseudomembranous	0	2 (6.7)	0	1 (3.3)
Erythematous	0	0	0	1 (3.3)
Angular cheilitis	3 (10)	5 (16.7)	2 (6.7)	1 (3.3)
Parotid enlargement	0	0	1 (3.3)	0
Herpes labialis	2 (6.7)	1 (3.3)	0	0
Linear gingival erythema	2 (6.7)	0	0	0
ANUG	0	1 (3.3)	0	0
Atypical oral ulcers	3 (10)	1 (3.3)	0	0
Molluscum contagiosum	2 (6.7)	1 (3.3)	0	0
Non‐Hodgkin's lymphoma	0	0	0	1 (3.3)
Total	12 (40)	11 (36.7)	3 (10)	4 (13.3)

The oral lesion prevalence description omitted the inclusion of cervical ‐ or any other – lymphadenopathies to prevent the occurrence of bias. In total 24 children presented with lymphadenopathy (36.4%) in this study and 9 exhibited other co‐morbidities that also result in cervical lymphadenopathies, such as cytomegalovirus infections (67%) and tuberculosis (15%). Similarly, a submental lymphadenopathy case in one patient could also be interpreted as bias. This child suffered from concomitant ulcerative infections and multiple carious teeth, which made it difficult to determine the exact cause of the lymphadenopathy. Acute necrotizing ulcerative gingivitis (ANUG) and acute necrotizing ulcerative periodontitis (ANUP) had a prevalence of 1.5%. Without definitive diagnostic aids to determine the exact diagnosis of the ulcer, a 6.1% prevalence of oral ulcerations was found in this study. Herpes labialis and molluscum contagiosum were diagnosed in 4.5% of the patients and Herpes Simplex in 1.5%. One child developed a NHL.

## 4 DISCUSSION

A comprehensive oral examination is an important clinical assessment tool for all oral health care workers. Evaluating oral soft tissue facilitates the recognition of oral mucosal lesions; this enables oral health care workers to provide the appropriate treatments or recommend further interventions and investigations geared toward proper diagnoses and treatments (Agbelusi & Wright, 2005). When targeted by a comprehensive extra‐ and intra‐oral examination, cervical lymphadenopathy is often detected in numerous patients. Studies that included cervical lymphadenopathy as part of their prevalence percentage concerning orofacial findings reported that the prevalence of “oral manifestations” ranged between 64% and 78% (Kaul, David, Savitha, Rema, & Ramnarayan, 2009; Nabbanja, Gitta, Peterson, & Rwenyonyi, 2013; Rwenyonyi et al., 2011). In this study, 36.4% of the sample participants presented with lymphadenopathy. Therefore, it was essential to exclude cervical lymphadenopathy from the prevalence percentage concerning oral mucosal lesions, as it is neither an oral lesion nor is it included among the orofacial lesions determined by Ramos‐Gomez et al. (1999) that are associated with pediatric HIV infection. Moreover, including cervical lymphadenopathy would have skewed the oral mucosal lesions data in the literature (Kaul et al., 2009; Nabbanja et al., 2013; Rwenyonyi et al., 2011). In this study, several children that exhibited other co‐morbidities that also result in cervical lymphadenopathies, such as cytomegalovirus infections and tuberculosis, were evaluated to ensure no bias occurred. Accordingly, the oral lesion prevalence description omitted the inclusion of cervical—or any other—lymphadenopathy.

The prevalence of HIV‐associated oral manifestations in children varies significantly among resource‐limited countries. The results from this specific South African population exhibited a prevalence of oral mucosal lesions (31.8%), which correlated well with the 31.4% prevalence in Brazil (Miziara & Weber, 2008). Conversely, several studies reported low prevalence rates: 8.3% in West Africa (Meless et al., 2014), 12.2% in Uganda (Rwenyonyi et al., 2011), and 13.3% in Mozambique (Sales‐Peres. et al., 2012). However, these findings are considerably lower than those in studies where large numbers of the participants were not on HAART medication, such as the following: Tanzania (43.1%) (Hamza et al., 2006), Nigeria (41.7%) (Olaniyi & Sunday, 2005), Uganda (48.9%) (Nabbanja et al., 2013), India (50%) (Kaul et al., 2009), South Africa (51.8%) (Duggal et al., 2010), and in Nigeria with 55.9% (Oladokun et al., 2013) and 61.9% (Adebola et al., 2012).

Oral candidiasis is a good indicator of immune suppression and is, therefore, commonly seen among patients who have progressed to AIDS (Coogan et al., 2005; Gaitán‐Cepeda et al., 2010; Nittayananta, 2016). In this study, 40.9% of the children exhibited some form of Candidiasis, while similar studies recorded a prevalence of 22.5%–83.3% (Gaitán‐Cepeda et al., 2014; Naidoo & Chikte, 2004; Samaranayake, 1992). Although pseudomembranous candidiasis was reported as the most prevalent variant among children, with values ranging between 6% and 72% (Nabbanja et al., 2013; Oladokun et al., 2013; Samaranayake, 1992), only 4.5% of this study's patients presented with this variant.

While parotid enlargements are rarely seen in adults, parotid‐ and other salivary gland enlargements are extremely common in children with HIV infections (Ramos‐Gomez et al., 1999); a study recorded a prevalence ranging between 0% and 58% (Schiødt et al., 1992). The recorded 1.5% prevalence of parotid enlargements found in this study is analogous with the results of studies conducted in Tanzania, India, Uganda, Nigeria, and West Africa (Hamza et al., 2006; Kaul et al., 2009; Meless et al., 2014; Oladokun et al., 2013; Rwenyonyi et al., 2011).

The only child in this study who did present with a parotid enlargement exhibited a high viral load. This finding correlates with the conclusions of several studies that assert that HIV‐associated parotid enlargements occur late during the infection, thus signifying a progression toward AIDS and immune failure (Jetpurwala & Jain, 2012; Prabhu R. V., Prabhu, V., Chatra, L., & Shenai, P., 2013).

The prevalence of linear gingival erythema (LGE) in children with HIV varies between different populations and can range anywhere between 0% and 48% (Hamza et al., 2006; Kaul et al., 2009; Ramos‐Gomez et al., 1999; Rwenyonyi et al., 2011). In this study, 3% of the patients had LGE and exhibited a viral load <50 copies/ml. Studies have linked the occurrence of gingivitis and periodontal disease in HIV‐infected individuals to other relevant factors, such as poor oral hygiene (dos Santos Pinheiro et al., 2009; Robinson, 2002). However, several children had poor oral hygiene and increased dental plaque, which made it diagnostically difficult to distinguish between LGE and conventional gingivitis. The low result of 3% is peculiar, as other studies comparing the prevalence of LGE in HIV‐infected and HIV‐negative participants found that LGE was not only equally common in both groups but was significantly associated with dental plaque in both groups as well (Grbic et al., 1995; Robinson, Sheiham, Challacombe, Wren, & Zakrzewska, 1998). This disparity suggests that LGE may be indistinguishable from conventional gingivitis, which would explain the wide variability of its prevalence (Robinson, 2002).

Acute necrotizing ulcerative gingivitis (ANUG) and acute necrotizing ulcerative periodontitis (ANUP) are far less common in children, with a low prevalence varying between 0.2% and 5% (Ramos‐Gomez et al., 1996; Ramos‐Gomez et al., 1999; Ranganathan & Hemalatha, 2006). Meless et al. (2014) found a prevalence of 1.7% concerning these diseases, which corroborates the 1.5% prevalence found in the patients of this study.

An atypical ulcerative lesion prevalence of 6% among outpatient children in South Africa (Naidoo & Chikte, 2004) corroborated the findings of this study. As ulcerative lesions often mimic each other, a definitive diagnosis could not be reached. Therefore, this study encapsulated all ulcerative conditions together, except for recurrent aphthous ulcers and herpes labialis (Naidoo & Chikte, 2004)—a form of the Herpes Simplex Virus that exhibited a prevalence of 1.5%. Furthermore, although this study did not find any recurrent aphthous ulceration cases, several studies have reported a higher prevalence of recurrent aphthous ulcerations that range between 0.4% and 14.1% (Meless et al., 2014; Nabbanja et al., 2013; Oladokun et al., 2013; Rwenyonyi et al., 2011). Additionally, several studies have reported that the herpes simplex virus (HSV) infection prevalence among their participants was between 1.7% and 24% (Bodhade, Ganvir, & Hazarey, 2011; Flaitz & Hicks, 2003; Ramos‐Gomez et al., 1999; Rwenyonyi et al., 2011). In this study, 4.5% of the patients presented with herpes labialis.

Molluscum contagiosum proved the most common lesion with a prevalence of 4.5%. This is higher than the percentages observed in other studies: 0.9% (Flaitz & Hicks, 2003) and 3% (Ranganathan et al., 2006). This heightened prevalence maybe because most of these children live in crowded households and institutions (Brown, Janniger, Schwartz, & Silverberg, 2006; Naidoo & Chikte, 2004; Reynolds, Holman, Yorita Christensen, Cheek, & Damon, 2009).

HIV‐associated malignancies are generally uncommon in the pediatric population (Davidson & Eley, 2010). Nonetheless, most studies found Kaposi sarcoma (KS) to be the most common neoplastic lesion (Hamza et al., 2006; Nabbanja et al., 2013; Oladokun et al., 2013). However, in this study, one child (1.5%) had developed a NHL. This child exhibited an extremely high viral load despite being on HAART medication, thus signifying immune failure. NHL is usually only seen in the late stages of AIDS where CD4+ counts are below 100 cells/mm^3^ (Bajpai & Pazare, 2010). In the current population, antiretroviral treatments caused most patients to retain high CD4+ counts and low viral loads. This may explain the low prevalence rate of NHL.

To assess the factors associated with the occurrence of oral lesions among the patients, the level of their immunosuppression was assessed. A study previously hypothesized that the presence of oral lesions in patients with HIV coincides with a deterioration of their immune status—a low CD4+ count and high viral load (Baghirath et al., 2013). Therefore, a lower CD4+ count is expected to lead to a predisposition for oral lesions (Adebola et al., 2012; Sales‐Peres et al., 2012). Additionally, a CD4+ count and viral load, although measuring different aspects of immunity, should display an inverse correlation (HIV i‐base, 2016). Furthermore, several studies assert that a CD4+ count functions better as an indicator of disease progression than a viral load (Adebola et al., 2012; Bodhade et al., 2011; Rwenyonyi et al., 2011; Sales‐Peres et al., 2012). However, this study, along with several others, found little to no correlations between the presence of oral mucosal lesions and CD4+ counts or viral loads (Nabbanja et al., 2013; Shah, 2006) thus, the lowered CD4+ count was a poor predictor (Gaitán‐Cepeda et al., 2010).

This is because the lesions they presented with (angular cheilitis, herpes labialis, atypical ulcers, and Molluscum Contagiosum) are easily treated with improved home care. This study observed children with a viral load below 50 copies/ml doing well clinically and was properly adhering to their HAART medication. Despite the proper HAART observation, the children with a viral load below 50 copies/ml showed oral mucosal lesions in 24.2%, with no oral mucosal lesions for the other half. Congruently, the findings of this study are corroborated by the type of oral mucosal lesions observed among HIV‐positive patients in Nigeria and their correlation with the patients' specific clinical‐stage, viral loads, and CD4+ counts (Oladokun et al., 2013). Furthermore, a study exploring the factors influencing the distribution of oral mucosal lesions in children with CD4+ counts below 500 cells/mm^3^ revealed that they had significantly more oral mucosal lesions than those with CD4+ counts above 500 cells/mm^3^ (Rwenyonyi et al., 2011). Conversely, this study reports the same number of patients with and without oral mucosal lesions for CD4+ counts above 500 cells/mm^3^ (Table 1). Additionally, another study concerning South African children with HIV discovered an association between viral loads and CD4+ counts with the presence of oral mucosal lesions (Duggal et al., 2010). Therefore, the results of this study verify other findings that state that oral mucosal lesions are independently associated with immunosuppression (Meless et al., 2014). Consequently, these findings suggest that CD4+ counts and viral loads are not indicators of disease progression for children between the ages of 2 and 12.

## 5 CONCLUSION

Several oral manifestations of HIV were observed in a portion of the study population despite the use of HAART medication; the most common of these lesions were candidiasis infections (particularly angular cheilitis). However, the presence of oral mucosal lesions was not significantly correlated with the immunosuppression status of the patients. Based on the results, CD4+ counts and viral load does not appear to be suitable markers of orofacial involvement in children. Nevertheless, the early recognition of oral mucosal lesions can still facilitate appropriate and early interventions for collaborative health platforms. This can only be accomplished through improved oral screening and referral processes between health practitioners.

### 5.1 Recommendations

This study highlights the need for oral health awareness and access to oral health services for children living with HIV in South Africa. Most of the patients' caregivers were unaware of services offered at Tygerberg Dental Faculty, UWC, as well as the importance of managing oral mucosal lesions. Therefore, education programs and primary health care screening programs should be implemented by the dental faculty to drive this service.

The research data also stressed the need for a collaborative effort between the PIDC and pediatric dental clinics to provide effective oral health services for children living with HIV.

### 5.2 Limitations of the study

A potential limitation of this study could be the small sample size at the PIDC, since this clinic is located in one of the nine provinces of South Africa. Larger sample sizes would have been more representative of the overall oral health status of children in South Africa.

## CONFLICT OF INTEREST

The authors declare no conflicts of interest.

## AUTHOR CONTRIBUTIONS

Riaan Mulder was responsible for writing the manuscript, writing a review, editing, formal statistical analysis, calibration of the data curation, as well as data curation oversight and blinding. Olorato Mathiba handled the conceptualization, investigation, data collection, experimental design, and writing of the original draft. Nadia Mohamed was responsible for the writing of the original draft, editing, and choosing the study methodology.

## ETHICS STATEMENT

Approval to conduct the study was obtained from the University of the Western Cape Research Ethics Committee (Project Registration Number: 15/6/83). The study conformed to the recognized standards outlined in the Declaration of Helsinki. All people in the study and their parents/guardians provided informed consent before they were included in the study. Patient anonymity was preserved, and all records were anonymized.

## Data Availability

Data available on request from the authors . The data that support the findings of this study are available from the corresponding author upon reasonable request.
